# Within-species variation of seed traits of dune engineering species across a European climatic gradient

**DOI:** 10.3389/fpls.2022.978205

**Published:** 2022-08-11

**Authors:** Silvia Del Vecchio, Shivam Kumar Sharma, Mario Pavan, Alicia Teresa Rosario Acosta, Gianluigi Bacchetta, Francesco de Bello, Maike Isermann, Richard Michalet, Gabriella Buffa

**Affiliations:** ^1^Department of Environmental Sciences, Informatics and Statistics, Ca’ Foscari University of Venice, Venice, Italy; ^2^School of Environmental Sciences, Jawaharlal Nehru University, New Delhi, India; ^3^Department of Sciences, University of Rome III, Rome, Italy; ^4^Sardinian Germplasm Bank (BG-SAR), Hortus Botanicus Karalitanus (HBK), University of Cagliari, Cagliari, Italy; ^5^Centro de Investigaciones sobre Desertificación (CSIC-UV-GV), Valencia, Spain; ^6^Lower Saxon Wadden Sea National Park Authority, Wilhelmshaven, Germany; ^7^UMR 5805 EPOC, University of Bordeaux, Talence Cedex, France

**Keywords:** climatic gradient, coastal systems, intraspecific variability, population, seed germination, seed mass

## Abstract

Within-species variation is a key component of biodiversity and linking it to climatic gradients may significantly improve our understanding of ecological processes. High variability can be expected in plant traits, but it is unclear to which extent it varies across populations under different climatic conditions. Here, we investigated seed trait variability and its environmental dependency across a latitudinal gradient of two widely distributed dune-engineering species (*Thinopyrum junceum* and *Calamagrostis arenaria*). Seed germination responses against temperature and seed mass were compared within and among six populations exposed to a gradient of temperature and precipitation regimes (Spiekeroog, DE; Bordeaux, FR; Valencia, ES; Cagliari, IT, Rome, IT; Venice, IT). Seed germination showed opposite trends in response to temperature experienced during emergence in both species: with some expectation, in populations exposed to severe winters, seed germination was warm-cued, whereas in populations from warm sites with dry summer, seed germination was cold-cued. In *C. arenaria*, variability in seed germination responses disappeared once the seed coat was incised. Seed mass from sites with low precipitation was smaller than that from sites with higher precipitation and was better explained by rainfall continentality than by aridity in summer. Within-population variability in seed germination accounted for 5 to 54%, while for seed mass it was lower than 40%. Seed trait variability can be considerable both within- and among-populations even at broad spatial scale. The variability may be hardly predictable since it only partially correlated with the analyzed climatic variables, and with expectation based on the climatic features of the seed site of origin. Considering seed traits variability in the analysis of ecological processes at both within- and among-population levels may help elucidate unclear patterns of species dynamics, thereby contributing to plan adequate measures to counteract biodiversity loss.

## Introduction

Within-species variation is a key component of biodiversity and of ecological processes (Albert et al., 2012; Al Hayek et al., 2015; Albert, 2015; Cochrane et al., 2015; Des Roches et al., 2018; Welles and Funk, 2021). However, this variability has often been neglected in plant species dynamics (e.g., species distribution and species coexistence), which were mostly analyzed by comparing averaged values of plant traits collected in different sites or also along wide geographical gradients (Funk et al., 2017; Del Vecchio et al., 2018). Indeed, the overlooking of within-species variation has been recognized as one of the factors producing not only uncertainty in predictive distribution models but also limited understanding of population dynamics and of species distribution patterns (Brun et al., 2020; Münkemüller et al., 2020; Westerband et al., 2021). Incorporating within-species variation in analyses of demographic parameters (Struckman et al., 2019), or of community structure (Bolnick et al., 2011) could significantly help to develop biologically realistic models and more accurate quantitative predictions of species trends.

The extent of trait variability depends on the trait considered and on the stage of plant development (Siefert et al., 2015; Funk et al., 2021; Welles and Funk, 2021). Traits that can be measured on both seedlings and adult plants (such as leaf mass per area, or water use efficiency) proved to have higher variability when measured on seedlings (Funk et al., 2021). Moreover, high variability can be expected in all those traits that are mainly driven by environmental cues (Walck et al., 2011; Cochrane et al., 2019; Catelotti et al., 2020). In this context, seed germination is a reproductive trait highly dependent on temperature, water, and nutrient availability (Walck et al., 2011; Baskin and Baskin, 2014); however, estimates of the extent of germination variability at the within-species level were underrepresented with respect to vegetative traits (e.g., specific leaf area, plant height; Westerband et al., 2021). Previous studies demonstrated that at species level, and at biogeographical scale, seed germination is related to macroclimate (mainly mean annual temperature and precipitation) probably to ensure the most favorable ecological window for seedling survival (Thompson, 1970; Allen and Meyer, 1998; Donohue et al., 2010; Rosbakh and Poschlod, 2015; Fernández-Pascual et al., 2019, 2021). As a result, different seed germination responses can be observed according to environmental variation (Fenner, 2000; Rosbakh and Poschlod, 2015), and species with similar geo-climatic origin (e.g., tropical species, or Mediterranean species) often share the same germination patterns (Fenner and Thompson, 2005; Dürr et al., 2015).

At the within-species level, different seed germination responses according to environmental variation were also observed (Fernández-Pascual et al., 2019; Picciau et al., 2019; Gremer et al., 2020). In sites with harsh winters (e.g., high latitudes or altitudes), several germination strategies protect seedlings from frost. Seeds from populations exposed to cold temperature are dormant and require a longer period of cold stratification to germinate in comparison to seeds from populations exposed to milder winters (Allen and Meyer, 1998; Cuena-Lombraña et al., 2018; Fernández-Pascual et al., 2019, 2021; Gremer et al., 2020); this strategy allows seeds to overwinter and germinate in spring. Accordingly, the base temperature for germination, i.e., the theoretical value of temperature at which germination can start, tends to be higher in sites where winter is the limiting season for survival, so as to assure germination would start when milder temperatures are sensed (Bürger et al., 2020).

At species level, germination is also regulated by the interaction between precipitation and temperature. Species base water potential, i.e., the theoretical value of water potential at which germination can start, increases at increasing temperatures (Dürr et al., 2015), indicating that species from warm sites avoid germinating under water stress. Moreover, cardinal temperatures for germination (base, optimum and ceiling germination temperatures) of species from regions with low amounts of precipitation, such as the Mediterranean region, are often lower than those of species from regions without dry seasons (Thompson, 1970; Fenner and Thompson, 2005). Such a mechanism promotes germination in autumn, thereby preventing seedlings from being exposed to summer drought. Some evidence of shifts in germination temperature in relation to water stress were also found at within-species level (Roy, 1982). Moreover, other effects of water stress were identified at within-species level such as variation of drought tolerance among populations (Cochrane et al., 2014; Chamorro et al., 2017; Gray et al., 2019), although tolerance to drought was not always correlated with precipitation gradients (Gray et al., 2019).

Seed trait variability was also observed for seed mass which shows a huge range of variation across species, and decreases at increasing latitude (Moles et al., 2007). In line with this pattern, species with large seed mass were found in warm sites (Murray et al., 2004; Moles et al., 2014). However, patterns of seed mass variation seem more complex, since seed mass is phylogenetically conserved (Arène et al., 2017), and is largely dependent on factors such as plant growth form and vegetation type (e.g., rain forest, desert, grassland, wetland; Moles et al., 2007). At the within-species level, seed mass variation was observed for several species (Vaughton and Ramsey, 1998; Del Vecchio et al., 2012; Cochrane et al., 2015; Soper Gorden et al., 2016; Zhang et al., 2020). However, the relationship between seed mass variation and climatic gradients, both, within- and among-species, still remains unclear (Soper Gorden et al., 2016; Garnier et al., 2019). Since large seeds produce seedlings more resistant to drought than seedlings from small seeds (Arène et al., 2017), a possible expected pattern is that larger seeds are favored in arid sites at the within-species level (Wang et al., 2020).

Despite environmental factors such as temperature and precipitation are expected to shape seed traits (Moles et al., 2007; Bürger et al., 2020; Fernández-Pascual et al., 2021), previous studies showed that seed trait variation at the within-species level does not always correlate with climatic variables. Seeds collected from populations exposed to different temperature and precipitation regimes had germination responses different than those expected on the base of the climatic variables of their site of origin (Cochrane et al., 2015; Finch et al., 2019), while seed mass variation at the within-species level was inconsistent among species (Soper Gorden et al., 2016). We still need broad-scale studies aiming to link within-species variability to climatic gradients in order to improve our understanding about which traits are more sensitive to environmental variation. Moreover, as the degree to which seed traits vary within species distribution range is mostly unknown (Kuppler et al., 2020; Westerband et al., 2021), these studies could be particularly useful to improve predictions of the impact of climate change on species dynamics and to elucidate the thresholds under which species will successfully establish if the environmental conditions change (Cuena-Lombraña et al., 2020).

In this regard, the study of widely distributed species which encompass contrasting environmental conditions could be particularly useful to clarify within-species variation and its environmental dependency. *Thinopyrum junceum* (L.) Á.Löve (= *Elymus farctus* (Viv.) Runemark ex Melderis; *Elytrigia juncea* (L.) Nevski) and *Calamagrostis arenaria* (L.) Roth (= *Ammophila arenaria* (L.) Link) are the main engineer species of European dune systems and occur along the coasts of the Mediterranean Basin up to the coasts of North European countries (Euro+Med 2006). Such wide continental distribution makes *T. junceum* and *C. arenaria* ideal model species to investigate within-species variation in seed traits. On these bases, the aim of this work is to test if germination patterns and seed mass vary consistently among populations, according to temperature and precipitation gradients at biogeographical scale. We hypothesized that seeds from populations occurring in warm sites with a dry season are adapted to germinate under colder cues and have larger seeds than populations from cold sites without dry season. Moreover, to investigate to what extent within-species variation is likely to be relevant at within-population level, or among-population level, for each species and for each population, we compared the contribution of within-population variability to the total variability.

## Materials and methods

### Seed collection and germination tests

**Figure 1:**
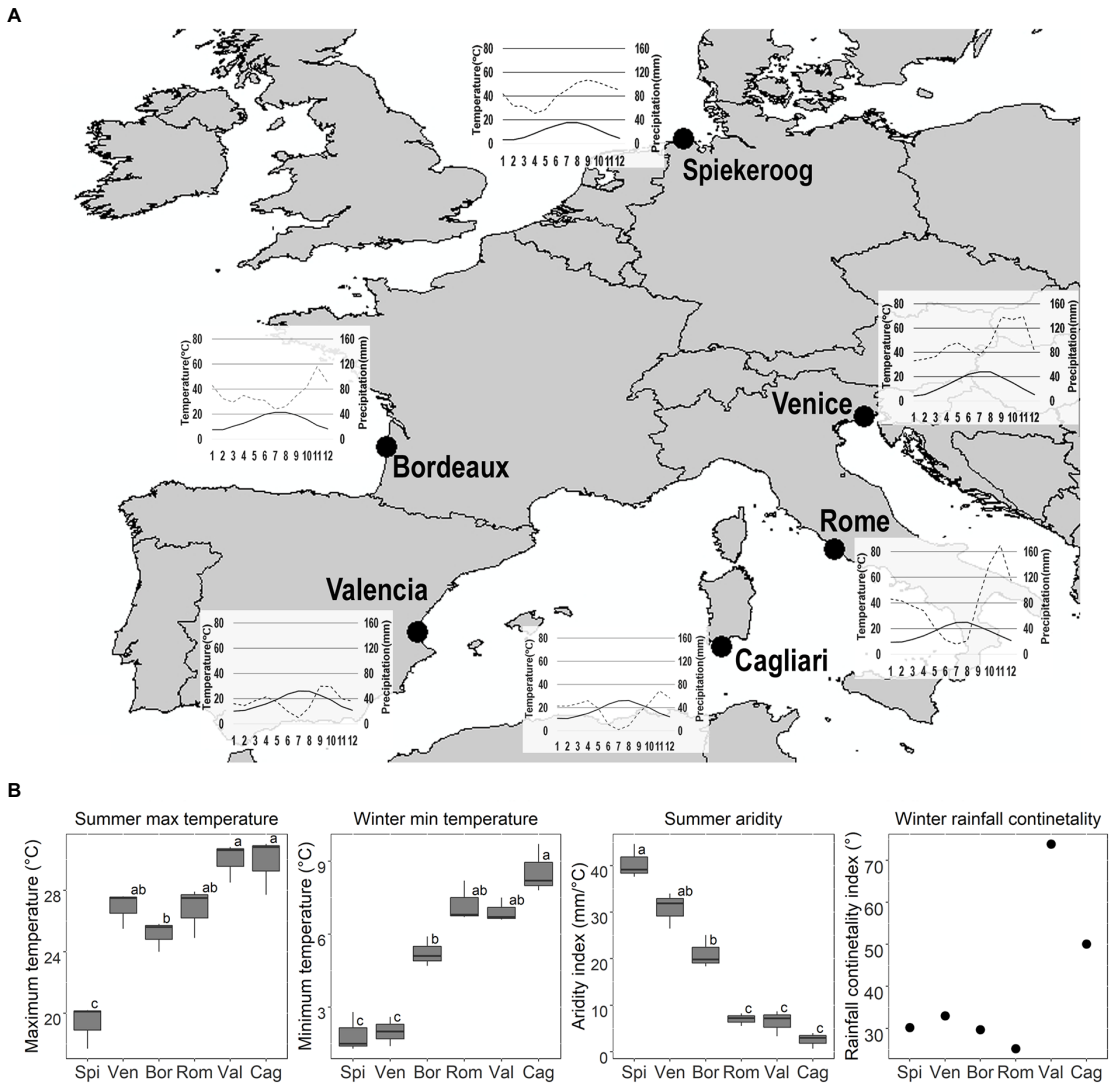
Seed collection sites, coordinates (Latitude and Longitude, in decimal degrees) and seed collection date: Spi = Spiekeroog (53.79360; 7.71150; 12/09/2019); Ven = Venice (45.69365; 13.08790; 24/07/2019): Bor = Bordeaux (44.76700; −1.20000; 21/07/2019); Rom = Rome (41.69210; 12.30090; 09/07/2019); Val = Valencia (39.36827; −0.30850; 08/08/2019); Cag = Cagliari (38.84522; 8.86276; 05/08/2019). Seeds of both species were collected on the same day, with the exception of *C. arenaria* from Rome, which was collected on 10/09/2019. Climatic data were sourced from www.Climate-Data.org, and from the available localities nearest to the sites of seed collection (for Spiekeroog: Spiekeroog; for Venice: Ca′ Ballarin; for Bordeaux: Lège-Cap-Ferret; for Rome: Fiumicino; for Valencia: Valencia; for Cagliari: Domus de Maria). **(A)** Thermopluviometric diagrams. Black line: temperature (T °C); Grey dashed line: precipitation (P mm). **(B)** Average summer (June–August) maximum T and aridity index, winter (December–February) minimum T, and rainfall continentality index for each site. High values of the aridity index indicate greater P regimes and/or lower summer T, thus, lower summer drought. Different letters indicate significant differences to the *post-hoc* Tukey tests.

Seeds were collected in six populations across the distribution range of the species, along the coasts of the Mediterranean Basin, Atlantic Ocean, and North Sea (Figure 1). Seeds were collected at time of dispersal (summer, see Figure 1 for collection date) on 50–100 randomly selected individuals at each site (ENSCONET, 2009). After collection seeds were sent to Ca′ Foscari University of Venice, where they were stored under controlled conditions (18°C, 40% RH) until the experiments started (storage time: 80 days for *T. junceum*, and 370 days for *C. arenaria*).

Imbibition tests, performed by comparing seed weight before and after 24 h of soaking in water, revealed seed coat water-permeability for both species. Germination conditions were based on germination tests and on pilot trials carried out on seeds from Venice from 2017 to 2019, aimed at exploring dormancy-breaking mechanisms for both species. Details are provided in Supplementary material S1.

For *T. junceum* the best germination was obtained in darkness and after cold stratification. Accordingly, for each site, four replicates of 20 seeds each were sown on 1% agar-water solution, and cold-stratified in darkness at 5°C for 3 months. Afterwards, seeds were moved at the constant temperature regimes of 10, 15, 20 and 25°C (in darkness).

*Calamagrostis arenaria* showed complex barriers to germination. Despite having implemented several dormancy-breaking mechanisms (Supplementary material S1), only chipping could successfully break dormancy. Thus, for each population, seeds were sown both without any pre-treatment, to compare the degree of dormancy across populations, and after chipping, to explore seed germination responses once dormancy has been removed. Chipping was carried out by gently and carefully removing with sandpaper a portion of seed coat close to the embryo for each seed before sowing. Both, non-chipped (integer) and chipped seeds were sown on 1% water-agar solution and incubated at 10, 15, 20 and 25°C (four replicates of 20 seeds per dish). Chipped seeds were incubated in a 12/12 h photoperiod, since pilot trials showed that dormancy was successfully broken in light conditions. Integer seeds were incubated in both a 12/12 h photoperiod and 24 h of constant darkness to investigate possible effects of darkness, since from the pilot trials we could not detect dormancy-breaking mechanisms and consequently the best germination conditions for integer seeds (Supplementary material S1). For *C. arenaria* tests were carried out for all populations with the exception of Cagliari and Bordeaux populations, for which seeds were not enough.

For both species, tests were ended when germination did not occur for 15 consecutive days, and after a maximum of 50 days of observation. Non-germinated seeds at the end of the tests were classified as potentially viable (with firm and white embryo) or dead (when they collapsed if pinched gently or had grey/brownish embryo) by the cut test (Davies et al., 2015).

### Data analyses

To explore the climatic conditions of the sites of seed collection, temperature and precipitation values were extracted from Climate-Data.org (www.climate-data.org; relative to the period 1999–2019). Such values were used to generate Thermopluviometric diagrams, showing the relation between precipitation amounts and mean temperature, and to calculate the monthly aridity index. The monthly aridity index was calculated as the ratio between the monthly precipitation*12 and the monthly mean temperature + 10; low values (< 10) indicate strong aridity, while high values (> 50) indicate very humid condition (De Martonne, 1926). For each site we also calculated the winter rainfall continentally index, which allows to separate continental from oceanic sites (Michalet et al., 2021). The index is angle that varies between 0° and 90°, with low values indicating oceanic climates, and high values continental climates. The rainfall continentality index was calculated according to the formula Cotg (*α*) = [4P − ((900 − A) ∕ 100) × (4P ∕ 10)] ∕ A, corrected for elevation below 100 m (Michalet, 1991; Michalet et al., 2021). Then we compared collection sites according to the maximum temperature and the aridity index during summer (from June to August) and the minimum temperature during winter (from December to February), which may be limiting seasons for seedling survival (Fenner and Thompson, 2005). We used ANOVA followed by *post hoc* Tukey’s test, using summer maximum temperature, summer aridity index, or winter minimum temperature, as dependent variables, and the sites of collection as grouping variable. ANOVA assumptions were assessed by Shapiro test, and transformed if necessary. Continental and oceanic sites were defined based on a natural break in the distribution of the winter rainfall continentality index, which occurred between 33° and 50°.

To test the effect of the source population and seed germination responses to the range of tested temperatures, for each species we performed GLMs with binomial error distribution and logit link function, setting temperature as numeric variable and population as factor (R Core Team, package lme4; Bates et al., 2014). For tests performed on integer seeds of *C. arenaria*, we included in the model also the photoperiod as factor. Since in some cases, *T. junceum* showed high seed germination during cold stratification at 5°C, thereby making the replicates at 10–25°C not comparable to the others, replicates with less than seven remnant (non-germinated) seeds after cold stratification were excluded from the model.

For each species, we investigated differences in seed mass among populations, sites with and without dry summer, and between more or less continental sites through ANOVA test (on log-transformed data for *T. junceum*). Sites were classified based on the results obtained by the comparison of the summer aridity index and rainfall continentally index.

To estimate the relative extent of seed germination and seed mass variability within-population with respect to the total variability, we calculated the ratio of the within-population sum of squares over the total sum of squares. The relative contribution of within-population variability was expressed as percentage. Low values (near to 0) are given when within-population variability is lower than total variability, and indicate a high variability among populations, while high values (near 100) are given when within-population variability and total variability have similar values and indicate low variability among populations. For seed germination we compared the variability of germination at each tested condition.

## Results

### Sites and climatic variables

The sites differed in their summer maximum temperature (Anova; *F* = 22.7; *p* < 0.0001), winter minimum temperature (Anova; *F* = 42.8; *p* < 0.0001), and summer aridity index (Anova; *F* = 58.2; *p* < 0.0001). The sites showed a latitudinal gradient from Spiekeroog to Cagliari, in which summer maximum temperature and winter minimum temperature increased (Figure 1). Spiekeroog had the lowest summer maximum temperature than all other sites, while Venice, Bordeaux, and Rome were intermediate between Spiekeroog and all other sites. Spiekeroog and Venice had the most severe winter, while Rome, Valencia and Cagliari the warmest winter. The northern populations (Spiekeroog, Venice, and Bordeaux) did not have a dry summer while the southern ones (Rome, Valencia and Cagliari) had a Mediterranean summer drought. Valencia and Cagliari were more continental than the other sites (Figure 1). Higher continentality for these sites was given by low precipitation during winter.

### Seed germination of *Thinopyrum junceum*

During cold stratification at 5°C, occasional germination occurred in all populations, although with lower values (< 5%) in seeds from Spiekeroog, Bordeaux and Rome with respect to the other populations (Table 1). Germination during cold stratification was particularly high in seeds from Cagliari.

**Table 1:** Germination percentage (mean ± SD) of cold-stratified seeds (3 months at 5°C) of *Thinopyrum junceum* at the tested temperatures, and during cold stratification.

Population	After cold stratification				Overall	During cold stratification	10°C	15°C	20°C	25°C		5°C
Spiekeroog	41.7 ± 11.8	52.8 ± 19.7	53.1 ± 0.3	54.0 ± 18.0	50.4 ± 12.3	3.5 ± 6.0
Venice	85.5 ± 3.4	78.6 ± 6.8	77.1 ± 19.1	34.5 ± 11.2	68.9 ± 23.3	19.7 ± 10.4
Bordeaux	7.5 ± 5.0	17.5 ± 14.4	21.7 ± 11.8	25.9 ± 7.9	18.2 ± 11.7	1.6 ± 3.0
Rome	85.0 ± 12.9	92.4 ± 6.5	73.8 ± 11.8	35.0 ± 10.8	71.5 ± 24.8	0.6 ± 1.7
Valencia	87.2 ± 4.9	41.0 ± 21.1	55.4 ± 4.1	43.6 ± 9.7	56.9 ± 21.8	23.1 ± 21.7
Cagliari	57.0 ± 14.1	54.7 ± 15.6	69.5 ± 9.7	40.2 ± 2.7	57.5 ± 14.2	38.1 ± 22.7

Overall germination is the average across the tested temperature of cold-stratified seeds.

The source population, temperature, and their interaction significantly influenced seed germination after cold stratification (Table 2). Germination percentages showed opposite trends over temperature depending on populations. Germination decreased with temperature in the population facing the Mediterranean Basin, i.e., Venice, Rome, Valencia and Cagliari, and increased in Spiekeroog and Bordeaux.

**Table 2:** Summary table of the GLMs to test the effect of temperature and the source population on seed germination of the target species.

*T. junceum*		*Df*	Deviance	Resid. *Df*	Resid. Dev	Pr(> Chi)
	NULL			84	504.84	
	Population	5	244.941	78	222.39	< 0.0001
	Temperature	1	37.506	83	467.33	< 0.0001
	Population × temperature	5	88.585	73	133.81	< 0.0001
*C. arenaria* - integer		*Df*	Deviance	Resid. Df	Resid. Dev	Pr(> Chi)
	NULL			127	783.71	
	Population	3	384.06	122	344.45	< 0.0001
	Temperature	1	16.16	126	767.55	< 0.0001
	Photoperiod	1	39.03	125	728.52	< 0.0001
	Population × temperature	3	98.18	118	239.19	< 0.0001
	Population × photoperiod	3	18.66	115	220.53	0.0003
	Temperature × photoperiod	1	7.08	121	337.37	0.0078
	Population × temperature × photoperiod	3	7.28	112	213.25	0.0635
*C. arenaria* - chipping		*Df*	Deviance	Resid. *Df*	Resid. Dev	Pr(> Chi)
	NULL			64	215.26	
	Population	3	102.439	60	110.70	< 0.0001
	Temperature	1	2.119	63	213.14	0.1455
	Population × temperature	3	4.182	57	106.52	0.2424

For integer seed of *Calamagrostis arenaria*, also the effect of photoperiod was tested.

Germination percentage averaged across temperature for each population had the lowest values in Bordeaux and Spiekeroog (Table 1). Cut test revealed that the majority of non-germinated seeds were potentially viable, with the exception of seeds from Cagliari, in which about 40% of non-germinated seeds were dead (Supplementary material S2).

### Seed germination of *Calamagrostis arenaria*

The source population, temperature, and photoperiod, and their two-way interaction, significantly influenced seed germination of integer seeds (Table 2), which however considerably varied across populations (Table 3). Overall germination was very low in seeds from Venice and Valencia, while seeds from Spiekeroog and Rome could germinate up to 41.2 ± 28.4% at 25°C and 55.6 ± 10.1% at 15°C, respectively, without any dormancy breaking pre-treatment. Despite the fluctuating values, seed germination increased at increasing temperature in Spiekeroog, and decreased in Rome (especially in the light condition, between 15°C and 25°C). In Valencia and Venice, although germination was low across all temperatures, the lowest values were observed at the highest tested temperature (25°C). The percentage of potentially viable non-germinated seeds largely exceeded the percentage of dead seeds (Supplementary material S2).

**Table 3:** Germination percentage (mean ± SD) of *C. arenaria* at each tested temperature. Overall germination for integer seeds is the average across the tested temperatures and the photoperiod, while for chipped seed is the average across the tested temperatures.

		10°C	15°C	20°C	25°C	Overall
Integer	Spiekeroog	0.6±1.8	6.9±7.6	16.1±8.7	41.2±28.4	16.2±21.4
	Venice	4.9±6.5	7.8±7.8	8.4±6.5	0.7±2.0	5.5±6.6
	Rome	28.6±19.1	55.6±10.1	42.6±23.3	32.4±21.7	39.8±21.1
	Valencia	3.8±5.2	6.0±7.2	2.0±3.9	0.7±2.0	3.1±5.1
Chipped	Spiekeroog	95.0±4.1	100.0±0.0	97.5±5.0	100.0±0.0	98.1±3.6
	Venice	68.4±4.3	73.6±14.4	73.6±7.7	76.9±7.4	73.2±8.7
	Rome	91.2±11.8	98.7±2.7	79.0±7.3	94.7±4.5	90.9±10.1
	Valencia	80.4±17.5	79.3±15.0	84.7±13.9	85.6±14.2	82.5±13.9

Tests for Cagliari and Bordeaux could not be performed due to seed shortage.

Once dormancy was removed by chipping, only the population influenced seed germination (Table 2). Seeds germinated at all tested temperatures with high values (higher than 65%) in all populations, and across temperatures (Table 3). The few non-germinated seeds were for the most part potentially viable, with the exception of seeds from Venice, where the percentage of dead seeds (approximately 19%) was higher than the percentage of potentially viable seeds (approximately 9%).

### Seed mass

**Figure 2:**
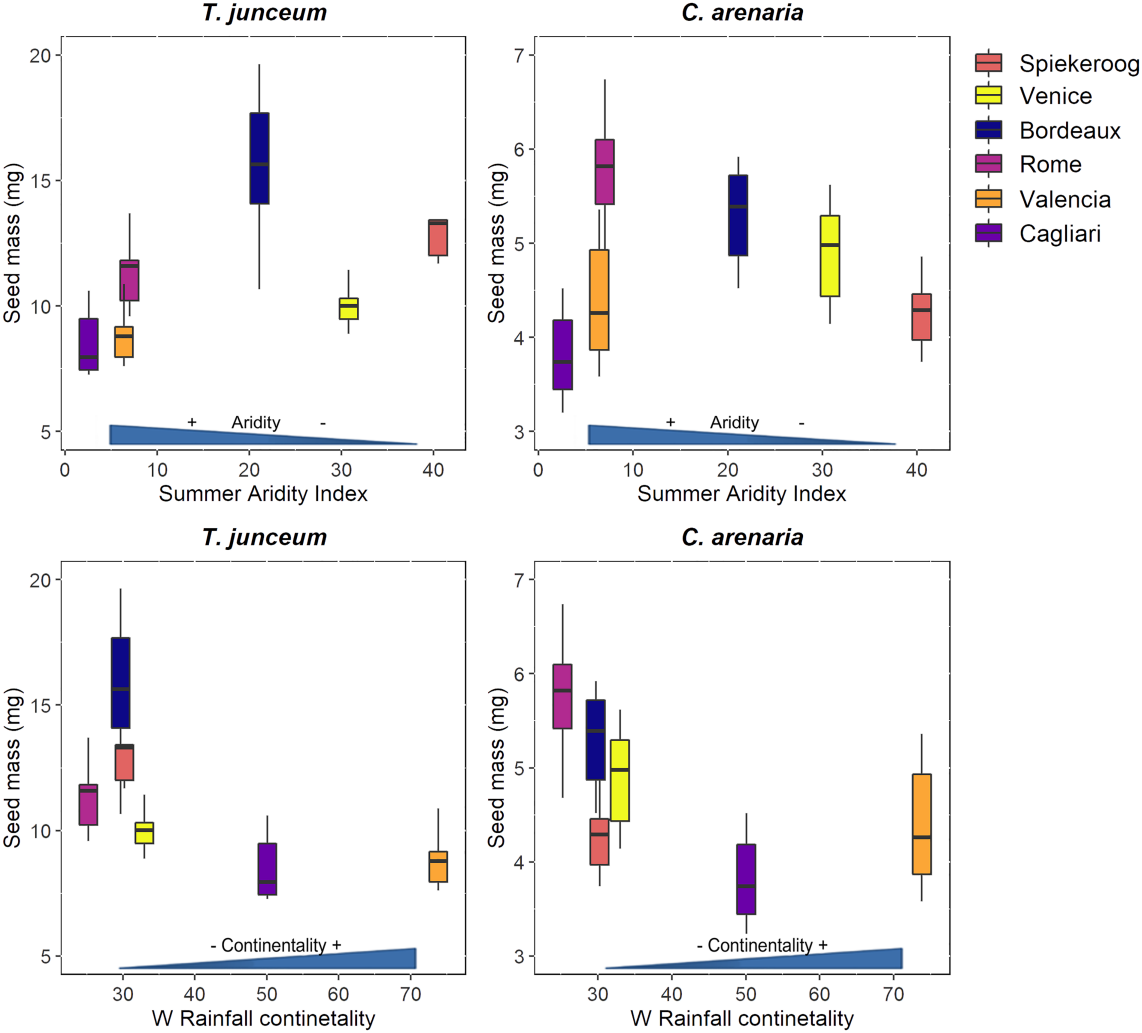
Seed mass of *Thinopyrum junceum* and *Calamagrostis arenaria* for each population. The summer aridity index and the rainfall continentality index of each population are shown on axis *x*. Color-blind-friendly palette was used for the figure (Garnier et al., 2021; Katsnelson, 2021).

Seed mass differed according to the population in both species (Table 4). For *T. junceum* the mass of seeds from sites with dry summer (Rome, Valencia, and Cagliari) and continental climate (Valencia and Cagliari) was smaller than the mass of seeds from sites without dry season (Spiekeroog, Venice and Bordeaux) and oceanic climate (Spiekeroog, Venice, Bordeaux, and Rome; Figure 2). For *C. arenaria* seed mass did not differ according to the summer aridity index, while seed mass from continental sites (Valencia and Cagliari) was smaller than in oceanic sites.

**Table 4:** *F* and *p* values of anova, comparing seed mass against the population, the summer aridity index, and the winter rainfall continentality index.

		*F* value	Pr(> F)
*T. junceum*	Population	33.67	1.93E-15
	Summer aridity index	30.2	9.10E-07
	Winter rainfall continentality index	49.88	2.30E-09
*C. arenaria*	Population	19.21	6.20E-11
	Summer aridity index	0.613	0.437
	Winter rainfall continentality index	24.62	6.45E-06

### Within- and among-populations variability

Overall, within-population variability had a high range of variation in seed germination, varying from 5 to 54% according to germination conditions, while for seed mass it was lower than 40%.

For *T. junceum*, within-population variability in seed germination accounted for 50% of the total variability during cold stratification at 5°C (Table 5). After cold stratification, within-population variability increased at increasing temperature. In *C. arenaria*, within-population variability had fluctuating values and showed no pattern against temperature (Table 5). Within-population variability was higher when seeds germinated in light than in darkness, and in chipped seeds compared to integer seeds.

**Table 5:** Ratio of within-population variability to total variability for seed germination at each tested condition and for seed mass.

	Within-population variability to total variability			
	*T. junceum*	*C. arenaria*		
		Integer		Chipped (%)
		Light (%)	Darkness (%)	
5°C	50%	–	–	–
10°C	7%	48	11	46
15°C	19%	14	8	38
20°C	21%	53	5	44
25°C	54%	13	11	40
Seed mass	25%	36		

Low values (near to 0) indicate high variability among populations (i.e., within-population variability is lower than total variability), while high values (near 100) indicate low variability among populations (within-population variability and total variability have similar values).

Within-population variability in seed mass was lower in *T. junceum* than in *C. arenaria* (25 and 36% of the total variability, respectively).

## Discussion

Seed germination responses against temperature of *T. junceum* and *C. arenaria* coming from different populations across their distribution range showed a strong differentiation according to seed provenance. However, seed germination responses only partially correlated with the latitudinal climatic gradient of the populations.

Concerning *T. junceum*, the requirement of a long period of cold stratification coupled with the adaptation to germinate at warm temperature observed in seeds from Spiekeroog and Bordeaux is clearly in line with the frozen-escape strategy (Billings and Mooney, 1968; Fenner and Thompson, 2005), since such germination requirements reflect adaptation to severe winter and absence of seasonal drought, that characterizes the sites of seed collection. Moreover, the overall germination across the tested temperature under 50% for seeds from Spiekeroog and Bordeaux, and the potential viability of non-germinated seeds indicated that seeds dormancy was only partially broken after 3 months of cold stratification (Allen and Meyer, 1998; Baskin and Baskin, 2014). It cannot be excluded that a longer cold stratification period could further promote the germination.

In agreement with adaptation to climatic variables, seeds of *T. junceum* from all the populations facing the Mediterranean Basin had a higher overall germination percentage after cold stratification, and, for seeds from Cagliari, Valencia and Venice, high germination during cold stratification at 5°C, thereby showing a lower degree of dormancy than seeds from Spiekeroog and Bordeaux (Allen and Meyer, 1998; Fenner and Thompson, 2005; Gremer et al., 2020). In addition to this, seeds were adapted to germinate at cold temperature, i.e., the opposite trend against temperature than seeds from Spiekeroog and Bordeaux. Such strategy of low degree of dormancy and adaptation to germinate at cold temperature is known as the “Mediterranean germination syndrome,” and has been reported for several Mediterranean species to avoid summer drought, allowing seeds to germinate in autumn, or after cold stratification at cool temperature of early spring (Ne’eman and Goubitz, 2000; Fenner and Thompson, 2005; Picciau et al., 2019).

An interesting result was found for seeds of *T. junceum* from Venice. This population showed the same drought-escape strategy of the other populations facing the Mediterranean Basin (Cagliari, Rome, and Valencia), although the climatic variables differed from those sites. These results from the Venetian seeds rises questions on how this species adjusted its germination in response to climatic pressure. The site has severe winter and lacks summer drought, as in Spiekeroog and Bordeaux, thus a frozen-escape strategy was expected rather than a drought-escape strategy. The cold stratification, required by the majority of seeds from Venice, likely allows seeds to overcome severe winter; however, it is not clear why seeds avoid germinating at high temperature if drought is not limiting. Since summer temperature in Venice resulted as high as in the other sites facing the Mediterranean basin tested in our research (see Figure 1), it could be hypothesized that the higher temperature experienced by seeds during maturation and dispersal is a cue to avoid germination in summer, regardless of the amount of precipitation. Accordingly, other authors have recognized that temperature during seed maturation has a crucial role in seed adaptation to environmental conditions (Fernández-Pascual et al., 2019).

Seed germination of cold- and warm-adapted populations can be influenced in different ways by the changing of environmental variables. Populations from both warm and cold sites may shift their germination timing in response of rising temperature, germinating earlier or later than they currently do, to match optimal germination conditions (Gioria et al., 2018; Fernández-Pascual et al., 2019; Porceddu et al., 2020). However, the germination of populations that need cold stratification may be impaired if the exposure to cold temperatures does not last enough to break dormancy (Allen and Meyer, 1998; Walck et al., 2011). In particular, rising temperature may affect those populations which require long periods of cold stratification to break dormancy.

As far as *C. arenaria* concerns, our results suggest that within-species variability in seed germination may depend on seed coat characteristics. Indeed, seed germination variability against temperature was observed in integer seeds, but not after that dormancy was broken by the incision of the seed coat. The seed germination niche was significantly expanded when dormancy was overcome, with the result that no germination trends were observed against temperature for all populations. Seed coat, although permeable to water, can impose dormancy in grasses (Simpson, 1990; Duclos et al., 2013; Baskin and Baskin, 2014), and in line with our results, in *C. arenaria* seed coat incision consistently broke dormancy very successfully, promoting seed germination at several temperature conditions (Van der Putten, 1990; Royal Botanic Gardens Kew, 2008). In integer seeds, similar to *T. junceum*, seeds from Spiekeroog showed the frozen-escape strategy, while seeds from Rome showed the drought-escape strategy, although with a less marked trend than in *T. junceum*. Thus, for this species environmental variables may play a major role during seed development rather than after seed dispersal, by influencing seed coat characteristics (e.g., thickness, permeability, concentration of germination inhibitors; Simpson, 1990; Fenner, 2000).

Our results on seed mass variability at the within-species level suggest that the effect of climatic variables on this trait is not clearly predictable. Seed mass varied across populations and was smaller in continental sites in both species, but decreased at increasing summer drought only in *T. junceum*. Despite large seed mass can be an advantage in sites with dry summer, since it provides seedlings a higher chance of survival under drought (Lloret et al., 1999; Wang et al., 2020), we found the opposite trend. Lower seed mass in drier conditions at the within-species level was found also for some species of Mediterranean scrub and desert, belonging to several families, including species from the Poaceae family (*Avena sterilis*, *Brachypodium distachyon*, *Crithopsis delileana*, and *Lolium rigidum*; Harel et al., 2011). Water stress experienced during seed development may reduce seed weight (Fenner and Thompson, 2005), but this is not always the case (Chamorro et al., 2016). Moreover, in *C. arenaria*, seed mass did not correlate with summer aridity, but it was related only to the rainfall continentality. For given species, rainfall continentality due to lower precipitation during winter could be a more important driver for seed mass than aridity in summer. Overall, our results support previous evidence that seed mass does not linearly correlate with climatic variables (Cochrane et al., 2015; Soper Gorden et al., 2016; Nunes et al., 2017).

Within-population variability in seed germination had a high range of variation, showing that it can be as important as, or even exceed, among-population variability. Despite at large scale, such as the European scale used in our research, within-population variability is expected to be lower than among-population variability (Siefert et al., 2015; Kuppler et al., 2020), our results suggest that for seed germination, general patterns of the relative contribution of within-population variability are difficult to be found. Since at given conditions it can represent about the half of total variability, considering within-population variability can help to clarify possible patterns when ecological processes are analyzed. Moreover, the contribution of within-population variability for seed mass differed between the two species, supporting the hypothesis that the degree of variation is species-specific, and in some cases, not negligible (Westerband et al., 2021).

## Conclusion

Our research demonstrated that at the within-species level the temperature thresholds for successful seed germination of the target species encompass a wide range, from 10 to 25°C. However, different populations may germinate under narrower ranges, having higher germination at cold or warm temperature, thereby having opposite germination trends against temperature. These results allow us to suggest that using averaged germination values at a given temperature is not always truly representative of the germination requirement of the species. Moreover, our research showed that seed mass could be better explained by rainfall continentality than summer aridity. As a general pattern, we found that within-species variability may be hardly predictable according to environmental variables, since seed trait variation may show different responses than those expected by analyzing the climatic features of the seed site of origin. To a large extent, our results support that research on intraspecific trait variability for wide-distributed species could provide important insights to reduce uncertainty prediction on local extinctions estimates, which could play an important role for planning adequate conservation and management actions to counteract biodiversity loss in relation to climatic changes.

## Data availability statement

The raw data supporting the conclusions of this article will be made available by the authors, without undue reservation.

## Author contributions

SDV and GBu conceived the research idea. SDV, SS, and MP performed the experiments. SDV, FdB, and RM analyzed the data. AA, GBa, FdB, MI, RM, and GBu provided significant comments on the first draft of the paper. SDV with contributions from all authors, wrote the paper. All authors contributed to the article and approved the submitted version.

## Funding

The Grant of Excellence Departments, MIUR-Italy (Article 1, Comma 314–337, Ley 232/2016) is gratefully acknowledged.

## Conflict of interest

The authors declare that the research was conducted in the absence of any commercial or financial relationships that could be construed as a potential conflict of interest.

## Publisher’s note

All claims expressed in this article are solely those of the authors and do not necessarily represent those of their affiliated organizations, or those of the publisher, the editors and the reviewers. Any product that may be evaluated in this article, or claim that may be made by its manufacturer, is not guaranteed or endorsed by the publisher.
