# Supplementary material for: Within-species variation of seed traits of dune engineering species across a European climatic gradient
Source: Front Plant Sci. 2022 Aug 11;13:978205. doi: 10.3389/fpls.2022.978205 (PMC9403325; doi:10.3389/fpls.2022.978205)
Supplement: Supplementary file 1 [file Table_2.DOCX]

Supplementary Material

# Supplementary Data S2

Germinated, potentially viable non-germinated seeds, and dead seeds at the end of the germination tests. Non-germinated seeds were classified as potentially viable or dead according to the cut test. Values represents averages across the tested temperature (10-25 °C).
