# Supplementary material for: Within-species variation of seed traits of dune engineering species across a European climatic gradient
Source: Front Plant Sci. 2022 Aug 11;13:978205. doi: 10.3389/fpls.2022.978205 (PMC9403325; doi:10.3389/fpls.2022.978205)
Supplement: Supplementary file 2 [file Data_Sheet_1.docx]

Supplementary Material

# Supplementary Data S1

Results of germination tests and pilot trials performed during 2017, 2018, and 2019 to explore dormancy breaking mechanisms for *Thinopyrum junceum* (L.) Á.Löve and *Calamagrostis arenaria* (L.) Roth. Tests were performed on seed from Venice (Italy). Seed were collected in the field on 100 randomly selected individuals and stored at 18°C and 40% RH before the experiments started. Germination tests were performed by sowing four replicates of 20 seeds each in Petri dishes, on 1% agar-water solution.

***Cold stratification***

In 2017 we performed an experiment to explore the effect of cold stratification on seed germination of both species (Tab S1). Seeds were incubated at 20°C in darkness after cold stratification (5°C) for periods of 0 (control), 1, 2, and 3 months. Cold stratification increased the germination percentage of *T. junceum* but couldn’t break dormancy in *C. arenaria* (Fig S1).

**Tab S1** Seed collection and storage time for each species

| **Species** | **Collection date** | **Seed storage time (days)** |
| --- | --- | --- |
| *Thinopyrum junceum* | 28/06/2017 | 110 |
| *Calamagrostis arenaria* | 27/07/2017 | 78 |


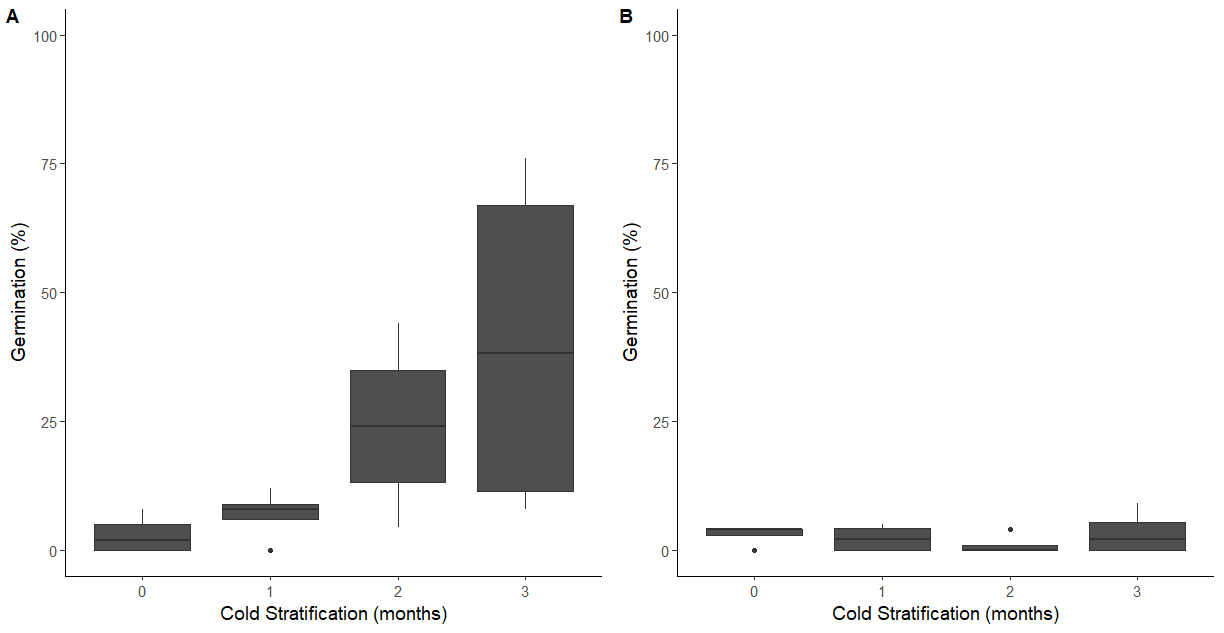


**Fig S1** (A) Germination percentage of *T. junceum* (A) and *C. arenaria* (B) at 20°C, after 0, 1, 2 or 3 months of cold stratification.

***Temperature and photoperiod***

In 2018 we performed germination tests to explore the effect of temperature and photoperiod on seed germination of both species (Tab S2). Seeds were incubated at constant temperature (from 5° C to 25° C), with a phoperiod of 12/12 h light/darkness and 24h darkness, without any pretreatment. *T. junceum* could germinate at all tested conditions, although, with higher values in darkness (Fig S2A). Conversely, *C. arenaria* had null or very low germination percentages at all tested conditions (Fig S2B). Germination was higher in darkness, although the maximum value was of 3.8 ± 4.15 % (at 20°C).

**Tab S2** Seed collection and storage time for each species

| **Species** | **Collection date** | **Seed storage time (days)** |
| --- | --- | --- |
| *Thinopyrum junceum* | 1/8/2018 | 77 |
| *Calamagrostis arenaria* | 4/7/2018 | 105 |

*
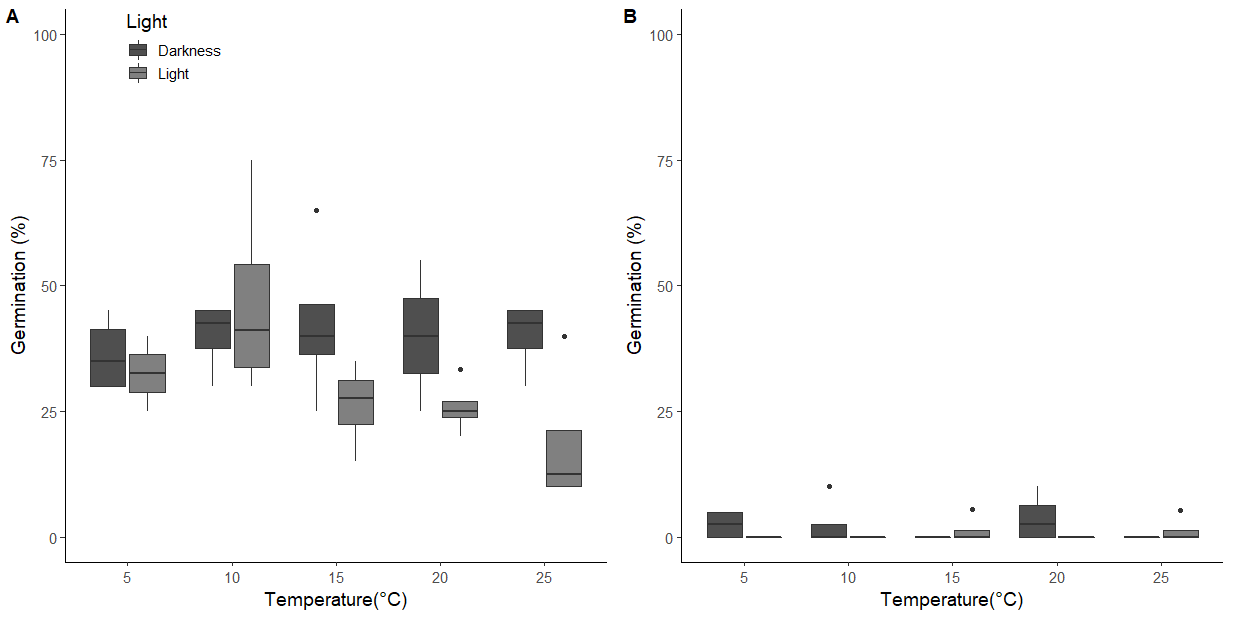
*

**Fig S2** Germination percentage of *T. junceum* (A) and *C. arenaria* (B) at different temperature and light regimes

***Other treatments***

Since *C. arenaria* didn’t germinate under any of the tested conditions, in 2019 we performed pilot trials (i.e. two replicates of 10 seeds each, sown on 1% agar) to investigate an array of dormancy breaking mechanisms, aimed at simulate the seasonal changes that seeds experience after dispersal. Seeds of *C. arenaria* were collected on 24/7/2019, and pretreatment started after 16 days of storage (at 18°C and 40% RH, as for the other seed lots used in the previous germination tests). To explore the effect of both constant and alternate temperature on seed germination, at the end of each pretreatment, seeds were exposed to the constant temperature of 20°C, and to the alternate temperature of 24/15 °C, in darkness. Temperatures represent the mean (20°C), the maximum (24°C), and minimum temperature (15°C) of September for Venice coast (averaged for the period 1994-2010, and for the climatic stations of Bibione, Cavallino-Treporti, Venice, Rosolina; Del Vecchio et al, 2021; <http://www.arpa.veneto.it>).

The pretreatments we performed were as follows:

*After ripening*: dry seeds were exposed to 24°C, in the light for 30 days, to simulate seed exposure to summer temperature in dry condition (e.g. on the soil surface). The temperature of 24°C represents the mean temperature in August for Venice (Del Vecchio et al, 2021; <http://www.arpa.veneto.it>).

*Warm stratification*: seed were sown on 1% agar-water solution, and exposed to 24°C in the darkness for 30 days, to simulate seed exposure to summer temperature in moist condition (e.g. seed buried in the soil).

*Cold stratification*: since cold stratification up to 3 months couldn’t break seed dormancy (Fig S1B), we tested the effect of a longer period. Accordingly, seed were sown on 1% agar-water solution, and exposed to 5°C, in the darkness for 4 months.

*After ripening combined with cold stratification*: dry seeds were exposed to 24°C in the light for 30 days. Successively they have been sown on 1% agar-water solution and exposed to 5°C in darkness for 4 months.

*Warm stratification combined with cold stratification*: seed were sown on 1% agar-water solution, and exposed to 24°C in the darkness for 30 days. Successively they have been exposed at 5°C in darkness for 4 months.

Germination percentage was very low at all tested conditions (Fig S3). The highest value was obtained by using after ripening followed by alternate temperature. However, the germination obtained under this condition was 20% (of the 10 seeds sown in the dish), thus such mechanism cannot be considered as successful for seed dormancy breaking.


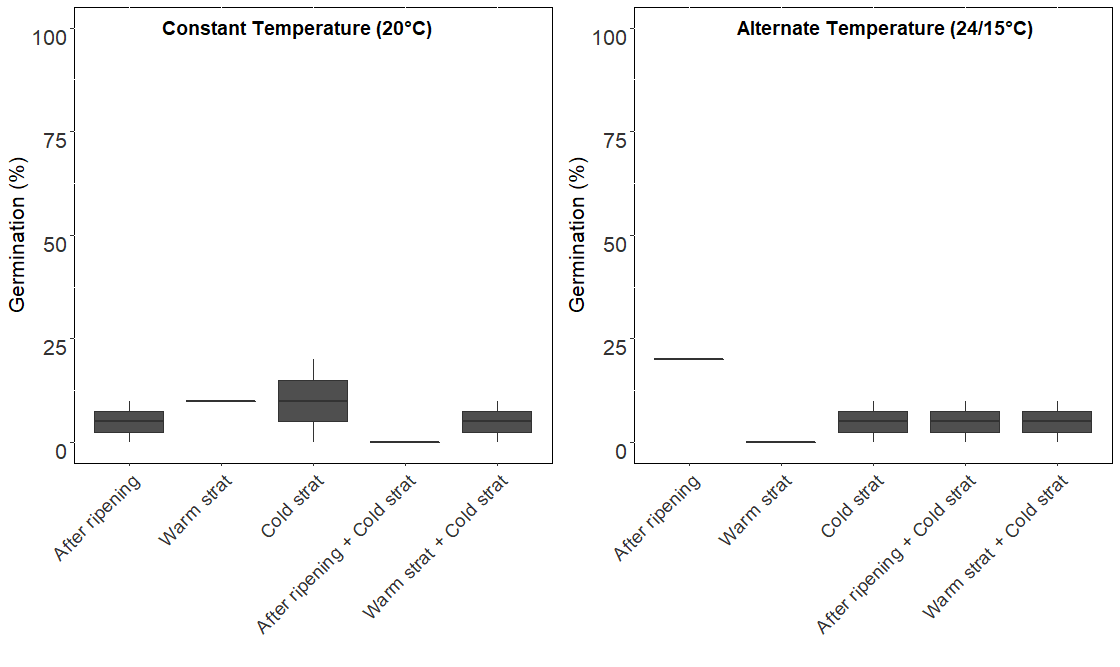


**Fig S3** Germination percentage of *C. arenaria* under different pretreatments and exposure to constant and alternate temperature.

**References**

Del Vecchio S, Mattana E, Ulian T, Buffa G (2021). Functional seed traits and germination patterns predict species coexistence in Northeast Mediterranean foredune communities. Annals of Botany 127: 361-370.
